# Psychiatry

**Published:** 1905-05-20

**Authors:** 


### PSYCHIATRY.

*(Continued from page* 123.)

**The Neuropathic Constitution.**—Congenital mental defect (idiocy and imbecility) depends upon arrested or defective development of the brain. In many cases this is of early embryonic origin, correlated often with well-defined physical malformations, which are known as anatomical "stigmata of degeneracy." These are met with abundantly in idiots and imbeciles of various classes. With regard to dementia præcox, we have seen that 70 per cent. of the subjects are hereditarily predisposed; that some of them are congenitally weak-minded, and that many of them owe their malady to the adjuvant effect in the first instance of such infections as scarlet fever, typhoid fever, phthisis, and other similar diseases. The well-known association of cretinoid idiocy and cretinoid imbecility with deficiency of the thyroid, and the relation of cretinism to myxœdema, have served to call attention to the importance of the "internal secretion" of organs in maintaining the normal development and growth of brain and body alike. It is suggestive, that causes which produce many of the forms of toxicopathic confusional insanity also produce organic or functional disease of the thyroid gland, the pituitary body, and possibly other ductless glands and organs. In the elucidation of the causes of insanity we have to do not with hereditary imperfections of the cerebral nervous system alone, but with bodily (visceral) changes which are equally important. What we call the neuropathic constitution must imply three things: (*a*) a developmental variation from the normal paths of cerebral evolution, implying a gross difference in the structure, composition, and connections of the nervous and accessory elements (non-nervous) of the central nervous system; (*b*) the variation in the anatomical form and physiological function of various bodily organs (stigmata of degeneracy) correlated thereto; and (*c*) a diminished power of immunity on the part of the body tissues against invasions by micro-organisms and toxins which are specially inimical to the life of the nervous system. Now all these features must be correlated with the physical conformation. The question then arises, says Maudsley,[2] as to how the neuropathic constitution is created, and how it is transmitted. If it were desired to breed a neuropathic degenerate being, with tendencies and instincts towards insanity, vice and crime, what would be the safest recipe? To engage his progenitors in an anti-social and immoral life, to impregnate them thoroughly with the poison of alcohol and syphilis, to make them lead lives of gluttony, wantonness, and sexual intemperance, to place them in an unhygienic environment. These factors would in time breed vicious, criminal, or insane degenerates, whether it be among the higher ranks of social life or in the slum-dwelling population. When mankind has learnt the way by which the degenerates and neuropaths have come to be it will be able to lay down rules to prevent their production in time to come, "but in order to do that it must substitute for the notion of *sin* and its consequences in a life to come . . . . the notion of *fault* of organic manufacture and its consequences from generation to generation in the life that now is." The cretin begot and born in the valleys of Savoy and Tyrol is as much the product of an unhygienic environment and of the intermarriage of parents similarly affected by such environment, as the idiot, the imbecile, the deaf mute, and the feeble-minded are the product of the complex conditions which constitute the slum life among the submerged tenth of our great cities. The neuropathic constitution or taint is allied on the one hand to the insane, and on the other to the criminal temperament. As crime and madness are both anti-social products of degeneracy, the aim of a fruitful inquiry must be in each particular instance—criminal, neuropathic, or insane—to discover its origin in the family stock, to uphold its relations to the circumstances of life, and to trace its dissemination, growth, and decay from generation to generation. Tramps and vagrants, prostitutes, and congenital criminals of the neurasthenic type (Benedikt) are also to be counted among the antisocial products of degeneracy, not alien enough in mind to be reckoned "dangerous" in the sense that lunatics are, but alien in morality and anti-social in conduct sufficiently to render them a burden to the State, a danger to its well-being, and a drain on its resources. Many cases of mild dementia præcox are met with, says Bonhöffer,[3] among tramps, vagrants, and prostitutes. The neuropathic character of the group is also shown by the tendency to the epileptic and explosive neuroses which are largely prevalent among the same. A struggle between social habits and savage instincts occurs seldom in their life experience, for their social habits are feeble and ill-developed, their savage instincts potent and predominant from a long line of degenerate or depraved ancestors. It is not possible to draw a distinct line of demarcation between insanity and vice; the neuropathic constitution unites the two, either when we have to deal with them socially as events, or when we investigate their causation in a scientific spirit.

[2] Pathology of Mind: 1895, p. 76–77. [3] Monatschr. für Psychiatr. und Neurol, 1898.

